# Communicating the risk of psychiatric in-patient or enhanced community care in dementia

**DOI:** 10.1192/bjo.2026.11011

**Published:** 2026-04-07

**Authors:** Maja Swirska, Sabina London, Emma Wolverson, Benjamin R. Underwood

**Affiliations:** Department of Psychiatry, University of Cambridge, UK; NHS Greater Glasgow and Clyde, Glasgow, UK; School of Medicine, University of Connecticut, USA; Geller Institute of Aging and Memory, University of West London, UK; Department of Psychiatry, Cambridgeshire and Peterborough Mental Health Partnership NHS Trust, Cambridge, UK

**Keywords:** Dementia, risk communication, PPIE, qualitative

## Abstract

**Background:**

Around 10% of people living with dementia (PLWD) will experience a deterioration in their condition that will require urgent (‘crisis’) community support or an in-patient psychiatric admission. We have previously shown that people at high risk of a crisis event can be identified at the point of diagnosis. How the idea that someone is at increased risk of experiencing a crisis event in their care is best communicated is not known. Here, we describe the analysis of interviews with clinicians and PLWD, to understand their perspective and the co-production of tools designed to support risk communication.

**Aims:**

To explore multistakeholder perspectives (healthcare professionals, carers, PLWD; *n* = 12) on the communication of risk of future in-patient psychiatric admission or enhanced ‘crisis’ community care and develop tools to support communication.

**Method:**

This pilot study used an experience-based, co-design approach. Reflexive thematic analysis was used to analyse transcripts, leading to co-development of draft educational materials.

**Results:**

We identified five themes that inform how risk should be communicated. Participants underlined the importance of timing, setting and follow-up appointments. Digital tools were considered essential, but they were not seen as a substitute to face-to-face appointments. Individual preferences varied, highlighting the need for patient-centred communication. The findings led to the co-development of clinician guidelines and educational materials.

**Conclusions:**

Risk communication in dementia is complex and must be personalised to be most effective. Our findings may have relevance for the communication of other areas of risk in dementia beyond risk of care crises.

The most common model for memory assessment services in the UK is for people to be seen, diagnosed and receive post-diagnostic support before being discharged back to their general practitioner (GP).^1^ People receiving services in this model may only return to specialist services at a point of crisis, an event incredibly distressing for both people living with dementia (PLWD) and carers,^2^ much later in the course of their condition. In previous work, we identified predictors for unplanned ‘crisis’ admission to psychiatric hospitals or intensive psychiatric care in the community.^3^ Through retrospective analysis of over 25 000 patient records, we have shown that up to 16% of PLWD in Cambridgeshire and up to 2.4% in Greater London subsequently deteriorate and require support, and that predictors of such crises can be identified at the point of diagnosis, including demographic and clinical factors such as age, marital status, Health of Nation Outcome Scales scores and cognitive performance.^3^ This raises the possibility of using these parameters to inform the design of a risk stratification clinical tool such as an app thatcould be used to calculate individual risk in memory clinics, but the development of such a tool does not currently enjoy any funding. For any risk prediction model or supportive app to have genuine clinical utility, we must first understand whether PLWD, their caregivers and healthcare professionals would benefit from its use, and how best to communicate complex ideas of risk to a population that may experience cognitive difficulties. Strategies for communicating with PLWD have been discussed in recent reviews, highlighting the limited availability of research including PLWD,^4–6^ as well as lack of guidance for healthcare professionals on how to have those consultations.^7,8^ Therefore, this work aims to (a) identify key barriers and facilitators of communicating risk of unplanned psychiatric admission or enhanced community ‘crisis’ care to PLWD and family carers; and (b) identify the tools and guidelines that PLWD, carers and healthcare professionals find clinically useful when discussing risk.

## Method

This work was conducted as part of a broader Master’s thesis by the first author, and elements of this work are derived from it.^9^ All procedures contributing to this work comply with the ethical standards of the relevant national and institutional committees on human experimentation and with the Helsinki Declaration of 1975, as revised in 2013.^10^ All procedures involving human patients were approved by the School of Biological Sciences Research Ethics Committee of the University of Cambridge (approval number PRE.2024.082). Written informed consent was obtained from all participants before participation. All participants were reimbursed for their time, and contributions were in line with national public involvement guidance.

### Study design

This study used an experience-based co-design, in a series of interactive workshops, to explore multistakeholder perspectives on risk communication and co-develop tools and guidelines for use in memory clinics. Our approach followed core experience-based co-design stages: observation and preparation, engaging patients and staff and gathering experiences, co-designing of prototype materials and feedback and refinement.^11^ The traditional experience-based co-design includes six stages (or five stages for the accelerated pathway). This methodology was adapted to fit our research objectives and discuss preformed concepts regarding creation of an application to aid in risk communication, based on our previous research on identifying participants at high risk of needing psychiatric admission.^3,12^ Patient and public involvement and engagement (PPIE) participants were predominantly involved from the prototype-testing and refinement stage, although interactive workshops allowed us to gather in-depth perspectives of lived experiences and to incorporate these perspectives into the development of tools.

The reporting of this study followed EQUATOR Network guidance and was informed by the Standards for Reporting Qualitative Research checklist.

### Sample, recruitment and eligibility criteria

People with dementia (*n* = 2) and family carers (*n* = 4) were recruited via purposeful opportunistic sampling from existing PPIE groups, including a specialist in-patient dementia experience PPIE group, whose members have all experienced care crises resulting in admission to psychiatric wards. Participants were also contacted through the local Cambridgeshire and Peterborough NHS Foundation Trust PPIE lead and groups supported by voluntary charities (e.g. 3 Nations Dementia Working Group supported by the Alzheimer’s Society). The sample size of the group was informed by practical recommendations for working with PLWD and carers from the Alzheimer’s Society, although was limited by difficulties with recruitment of PLWD.^13,14^ Participants from these existing PPIE groups were emailed a participant information sheet (PIS), which included the relevant information regarding taking part and were asked to reach out via email if they were willing to take part. Written consent was obtained from all participants through electronic or handwritten signatures. Participants were also provided with an email to the Cambridgeshire and Peterborough NHS Foundation Trust PPIE Lead, who was independent of the research team, and was able to assist with any concerns.

We recruited healthcare professionals working in dementia care (*n* = 6; two doctors, two nurses and two clinical psychologists), through an informal national community of practice for in-patient dementia care. Healthcare professionals and PLWD were provided a PIS via email and invited to express their interest in participating.

Because we recruited via existing PPIE groups and informal professional networks, expressions of interest were self-initiated, and the total number of individuals approached or declining participation could not be quantified.

The participant demographic table is presented in Appendix 1.

#### Inclusion criteria for participants

To be included in the study, participants needed to have experience of UK dementia care, and the ability to participate in online meetings via Microsoft Teams. PLWD needed a formal diagnosis and prior use of dementia care services, whereas family carers needed to support someone with dementia who had used such services. Healthcare professionals (e.g. doctors, nurses, psychologists) required National Health Service (NHS) experience in dementia diagnostic care.

#### Exclusion criteria for participants

Participants who were unable to provide informed consent were excluded from the study.

### Data collection

Participants were invited to take part in three workshops, separating PLWD and carers (group 1) and healthcare professionals (group 2) during the first two workshops. Caregivers and PLWD invited to participate either jointly or separately, according to their personal preference. Separating groups allowed for sharing of specific experiences and perspectives without influence or pressure from other groups, and ensured tailored facilitation to groups specific needs and communication style. These workshops were held virtually, a format which has been successfully used for experience-based co-design methodology and dementia focus groups in the past.^15^ All workshops were conducted on Microsoft Teams and transcribed using its software (version 1.0.97 (2025 release) for Windows, Microsoft Coorporation, Redmond, WA, USA; https://www.microsoft.com/en-gb/microsoft-teams/download-app).

The first workshop introduced the concept behind identifying people at risk of care crises and the potential to use a clinical tool or app. This workshop served as the basis of understanding whether disclosing risk of future unplanned psychiatric admission or enhanced care services is viewed as beneficial by PLWD, caregivers and healthcare professionals. The aim of an application was discussed as a potential tool used for healthcare professionals to quantify the level of risk for each individual. This workshop also explored whether PLWD and caregivers would want to be able to access their clinical risk score directly. Additionally, two fictional case examples were created to trigger discussions about risk communication (with a high-risk versus low-risk scenario), but the conversation was ultimately guided by the experiences of participants.

Based on topics discussed in the first workshop, the second workshop focused on methods of risk communication. The materials incorporated into the workshops were informed by literature on the topic of risk communication in dementia, including a variety of different representations and visualisations of risk.^16–20^

Qualitative analysis of the first two workshops (four in total) shaped prototype guideline and material development, which were shared with participants ahead of the final workshop. A final shared multistakeholder workshop, which brought together PLWD, carers and healthcare staff, allowed for direct exchange of views especially with regards to the generated materials. Participants in the third workshop provided feedback and improvements on the draft guidelines and communication aids, which led to the creation of the co-developed guidelines and a written leaflet on how to disclose risk.

**Fig. 1:**
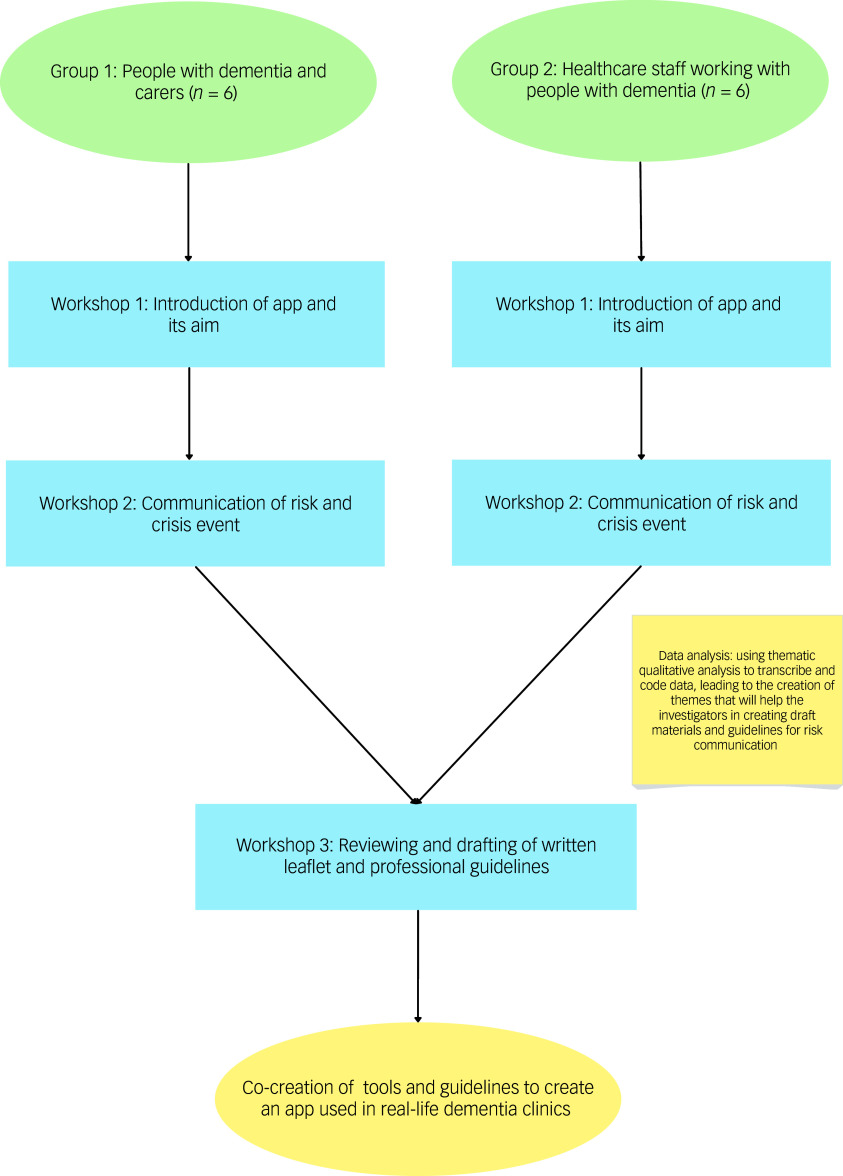
Study flowchart outlining the timeline of the workshops.

The workshop timeline and methodology are accurately outlined in Fig. 1.

### Data analysis

The transcribed data from the first two sets of workshops was analysed using reflexive thematic analysis (RTA), as developed by Braun and Clarke.^21^ This form of qualitative analysis was chosen as it reflects the aim of this work to understand risk communication and co-design effective tools to facilitate this. This allowed the researchers to find shared perspectives between study participants and embrace differences in participants’ experiences. The flexible approach offered by RTA allowed for the creation of meaningful conclusions shaped by the active influence of the researcher, which, ultimately, were part of the co-design process.^22^ Although the workshops were primarily designed by the research team, with the introduction of risk communication concepts and the idea behind the prototype application, we recognise that this framing may have influenced the directions of discussion. Nevertheless, the themes were co-constructed through the interaction between researchers and participants, reflecting not only the researchers’ initial framing, but also the perspectives and shared understandings that emerged during the workshops. Additionally, rather than seeking data saturation in the conventional sense, we assessed information power, as described by Malterud et al,^23^ acknowledging the small, relatively homogenous sample and treating the resulting themes as exploratory and hypothesis-generating.

As for the RTA process, one of the researchers (M.S.) carefully familiarised themselves with the data, re-reading transcript data and re-watching workshop recordings. Following familiarisation with the data, initial codes were generated. Semantic coding was prioritised in this work, to gather shared views and to guide the co-design process of this work, but latent meanings have emerged throughout the analysis of this work.^24^ The initial codes were then refined and restructured, leading to development of initial themes. Themes were then reviewed and reshaped by discarding and merging several themes, subsequently leading to the creation of subthemes. A second researcher (E.W.) was involved in collaborative coding and theme development, to support reflexive engagement with the data and minimise bias.

### Participatory co-design

Prototype clinician guidelines and a patient/carer leaflet on risk communication were developed by the research team following preliminary analysis. These were then refined collaboratively in the third workshop. Materials were explicitly presented as discussion tools rather than final products, and may be used as preliminary materials to guide further material development.

## Results

### Qualitative findings

The first question of the first workshop was whether clinicians, PLWD and carers would benefit from a prediction disclosing their future risk of psychiatric admission or needing enhanced care. All participants in group 1 expressed that they thought they would benefit from an application being introduced to memory clinics at the point of diagnosis. One of the carers said:

‘There might have been indicators that an app would have picked up for those sorts of incidents to indicate some more intervention of an earlier stage rather than wait for the final crisis, which led to hospital admission.’ (Caregiver, Participant 1)

Healthcare professionals in group 2 shared similar views, with all participants endorsing implementing a risk prediction app, but similarly, it would only be beneficial if there was a targeted intervention in place to support those at higher risk:

‘So, I would say it’s useful if there’s something that we can do about it. So, if our services reconfigure to allow us to keep these patients open to our services, that would be number 1.’ (Healthcare professional, Participant 9)

Following conversations surrounding the introduction of clinical risk disclosure supported by an app, we continued to discuss how to safely and effectively discuss risk between healthcare professionals, caregivers and PLWD.

**Fig. 2:**
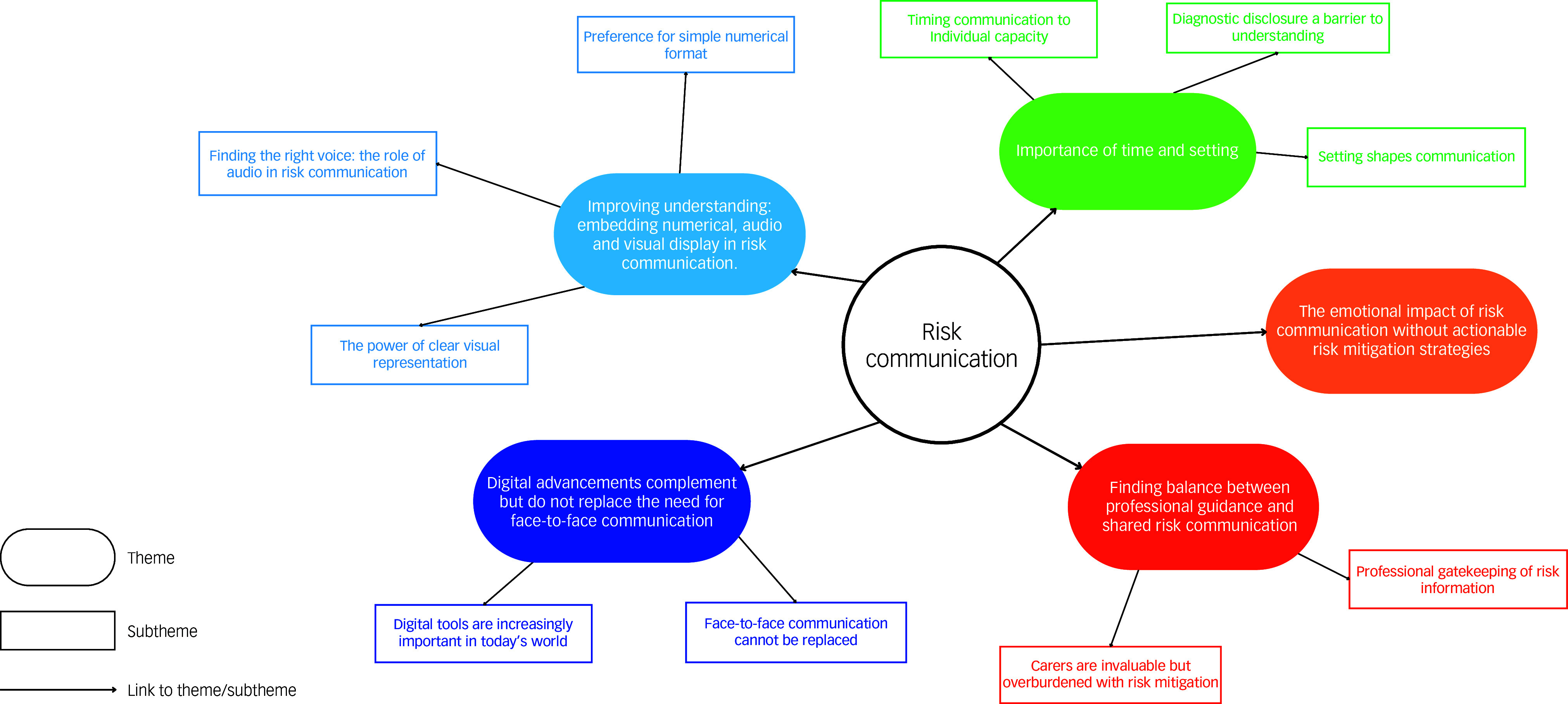
Finalised thematic map demonstrating five themes and relevant subthemes.

Five themes were developed following RTA of the first two workshops, encompassing a total of ten subthemes. The final thematic map is available in Fig. 2.

#### Theme 1: the importance of time and setting

In terms of time and setting, both groups emphasised that during the initial diagnostic appointment, it may be overwhelming for patients to receive risk information. There was a clear preference for communicating risk in the post-diagnostic setting as opposed to the appointment where a diagnosis was first disclosed. One of the caregivers expressed that:

‘It is actually getting the diagnosis that can be quite a lot to take in at that first session in the memory clinic or wherever and maybe, you know… there needs to be a follow up after a couple of months say. When both the person, the patient and the carer or family member of whoever is with them has had a chance to sort of think things through and that it would be easier.’ (Caregiver, Participant 3)

The ideal location of these conversations was also a topic of discussion, deciding whether home or clinics are best to disclose this information. One of the healthcare professionals supported disclosing research results in clinics:

‘I mean, I think clinic is a neutral space. Home is their turf, and so I think there is something about the neutrality of being in a clinic that sometimes does make it easier to have difficult conversations. I also think there’s something inherent there in the behavioural experiment of getting them to the appointment in the first place. That sometimes can be quite telling.’ (Healthcare professional, Participant 10)

#### Theme 2: finding balance between professional guidance and shared risk communication

Participants underlined the importance of shared communication between healthcare professionals, carers and PLWD, debating the complex dynamic during these conversations. Many healthcare professionals viewed carers as invaluable to risk communication, highlighting how risk communication is a collaborative process:

‘But obviously in dementia, generally most of the patients come with family members or carers. So, it’s a joint discussion with them.’ (Healthcare professional, Participant 11)

By analysing the data-set more latently, hidden perspectives from healthcare professionals suggested that they are often the drivers and instigators of risk communication. They were often the ones to decide what risks to disclose, as well as when and where to do so. One of the healthcare professionals said this when discussing dementia risk to those that are not ready to accept a diagnosis, proving how healthcare professionals are often the ones to decide what to discuss:

‘Reliably then it becomes apparent to you that there might be some problems, but it still doesn’t mean that you would be able to tell them that this is what I worry about, this is what I’m noticing, so there has to be some way of telling them that you have some concerns or maybe just skip that bit and say you know what I think you could do with a lot of help.’ (Healthcare professional, Participant 12)

Family caregivers’ and PLWD’ experience of risk communication was quite different, with participants reporting that they felt risk was not appropriately addressed in memory clinics, leaving them to try and understand their risk alone:

‘We were told initially that [Participant 4, PLWD], wouldn’t be able to drive any longer, but we’ve never really been told about any other risks. Particularly, we just work it out for ourselves.’ (Carer, Participant 3)

Carers also expressed that they often felt unsupported with risk management, did not feel listened to and felt they were not asked what risks they felt comfortable managing. One of the carers underlined how they were were not able to share their partner’s care preferences when they were admitted to hospital:

‘And when I tried to express it for him, I sort of felt I wasn’t heard...’ (Carer, Participant 2)

and expressed this when talking about risk management responsibilities:

‘No, I just took it on because he wouldn’t have been able to. I mean, I had issues at times.’ (Carer, Participant 2)

#### Theme 3: the emotional impact of risk communication without actionable risk mitigation strategies

Risk communication was viewed as emotionally charged, and healthcare professionals underlined how these conversations may be difficult to navigate, highlighting the importance of disclosing risk in a sensitive way:

‘Obviously it [discussing risk] will have to be done in a very sensitive manner…. and of course, we are going to deliver that as sensitively as possible.’ (Healthcare professional, Participant 11)

Discussions arose around communicating ‘high’ versus ‘low’ risk, with additional concerns around the emotional impact of communicating ‘high risk’, as it was often viewed as something scary and upsetting by PLWD and caregivers. One of the carers outlined how delivery of risk results should not differ between high versus low risk, but the emotional impact of risk communication may vary greatly between those two scenarios:

‘I think the only difference is the fear factor. Being on the receiving end about whether you know, you should be scared to death now, or maybe further down the line.’ (Caregiver, Participant 1)

A key solution for healthcare professionals for the dilemma of sensitively disclosing risk, seemed to be the introduction of risk mitigation that would offer patients hope for reducing their risk in the future:

‘And telling somebody you’re at high risk of, you know having a crisis event and then discharging you, there needs to be a sort of discussion because we’re concerned that you’re presented with a high risk of this. Then this is what we would like to suggest. This is how we’re going to try and mitigate this. This is how we’re going to try and work with you about this.’ (Healthcare professional, Participant 9)

#### Theme 4: improving understanding: embedding numerical, audio and visual display in risk communication

Our findings indicate that integrating numerical, audio and visual formats makes communication understandable by a larger proportion of patients. Multiple approaches should be used to communicate risk effectively, with one of the healthcare professionals saying:

‘I think if we’ve got multiple mediums for being able to help communicate this to people all to the better.’ (Healthcare professional, Participant 10)

However, in response, another highlighted that it is required by law to provide information in various formats:

‘Yep, I agree, I mean, it’s the law that we have to provide information in accessible ways. So actually, we don’t have any choice over this.’ (Healthcare professional, Participant 9)

**Fig. 3:**
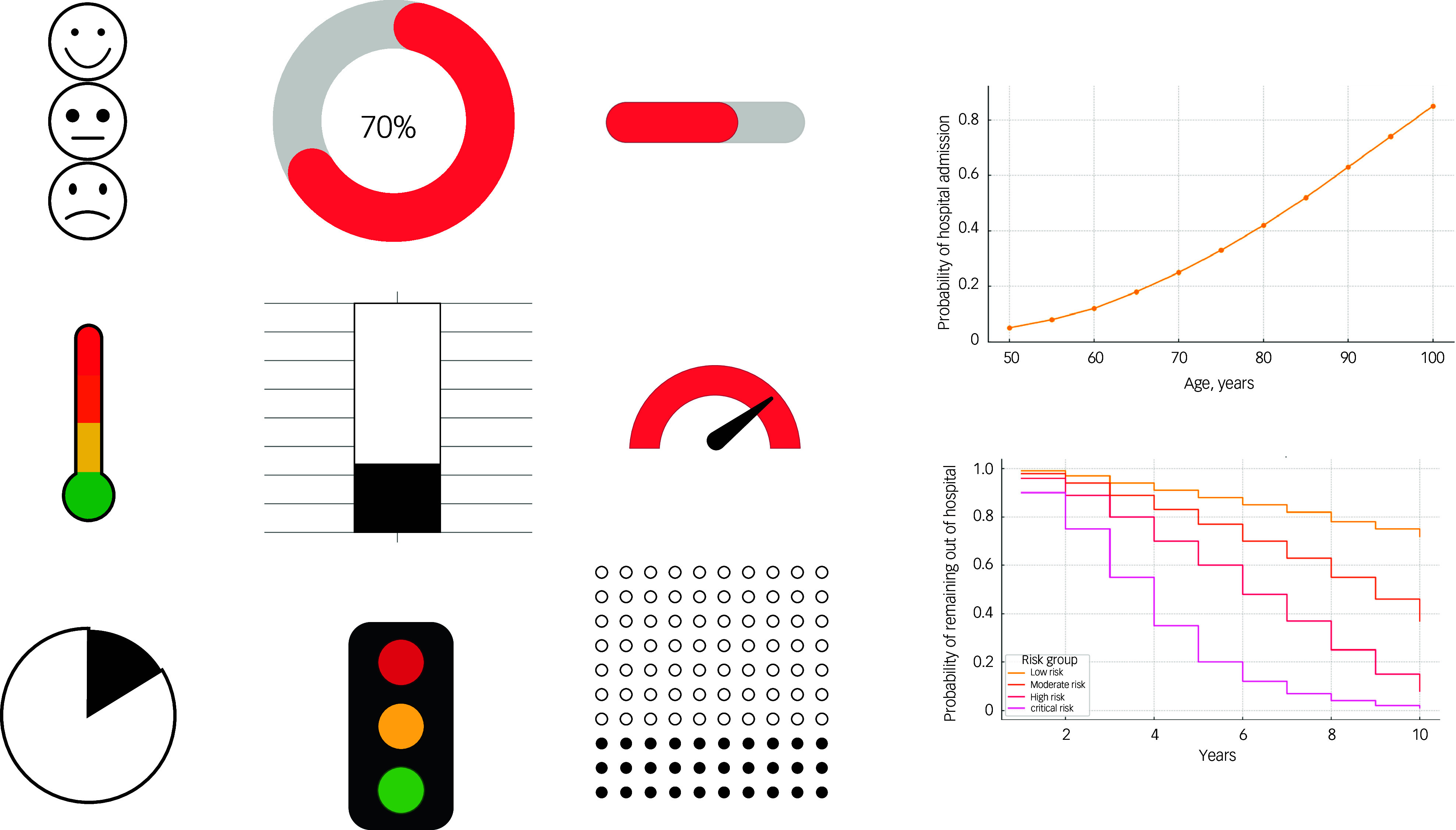
Examples of visual representation of risk used in workshops.

As part of the workshop, participants were presented with a variety of different visual ways of conveying risk, represented in Fig. 3. In terms of visual information, bar and pie charts were preferred, often described as the most clear and straightforward options, as opposed to risk icon arrays (often confusing to visually distinguish) or Kaplan–Meier graphs and line plots (too complex to understand). Participants most preferred a ’doughnut’ chart, a variation of a pie chart with a blank centre, as it visually represented their risk with a numerical percentage displayed in the middle:

‘I say what I see. Pie chart I get. I get them at the moment, but I don’t know whether I will later on. Bar graphs are easiest, I think.’ (PLWD, Participant 6)

Numerically, percentages, ratios out of a 100 and textual descriptors (high/medium/low) were preferred as opposed to decimals, fractions or ratios using other denominators:

‘I think the first two are sort of reasonably equivalent, to be quite honest [percentages and ratios]. That’s the sort of terminology you often see used in advertisements, and three out of 10 cats, or whatever it is, that kind of thing.’ (Caregiver, Participant 5)

Participants preferred voices that were familiar in accent, deeper, clear in articulation and slower in pace to aid in understanding:

‘The deeper the voice I think for me worked. You know, I think preferably for the four countries, their own accents.’ (PLWD, Participant 6)

#### Theme 5: digital advancements complement but do not replace the need for face-to-face communication

Mobile tools, websites and educational videos were welcomed by patients and caregivers and viewed as useful for reinforcing information throughout the initial risk discussion as well as at home, where they could be accessed at later time points. Throughout the workshops we asked whether a website would be a useful tool to convey risk information, to which Participant 4 (PLWD) replied ‘Yes’ and Participants 6 replied ‘It would. You’d have to make sure we knew it was a proper website and not whatever website. You know what I mean?’ (PLWD, Participant 6). The idea of the introduction of digital tools was also reinforced by healthcare professionals, who approved the introduction of an application if it meant it may prevent duplication:

‘Yeah, I think anything user friendly and simple is always helpful. So, for us, if it is, if the app or whatever we use is able to pull it [patient information] from our system would prevent duplication. I think a handheld device, I would agree maybe just be more user friendly and you do it there and then and it’s just a link on your phone.’ (Healthcare professional, Participant 11)

Participants, especially patients with memory difficulties, appreciated the idea of referring back to information learnt in consultations. Despite the strong support for digital communication and the potential introduction of an app, both healthcare professionals, caregivers and PLWD underlined the need for other forms of risk communication such as continued face-to-face appointments or written information. One of the carers highlighted the importance face-to-face consultations and take-home materials used together to deliver effective risk communication:

‘So, it was nice to have that initial face to face consultation, to be able to talk about the condition and then go home and look at the options in a bit more detail…you can then go back and digest it.’ (Carer, Participant 5)

### Co-design of materials

Following, qualitative analysis of the workshop transcripts, we developed preliminary materials, which included a guideline for clinicians and a written leaflet for PLWD and caregivers. These materials were shared with all stakeholder groups for feedback.

The developed materials are shown in Appendices 2 and 3.

The recommendations for clinicians (Appendix 2) included the following: (a) ensuring that communication is clear and sensitive with frequent checking of understanding; (b) tailoring communication to individuals’ abilities, circumstances and emotions; (c) actively involving relatives and caregivers; (d) using various visual and audio materials to enhance comprehension; (e) focusing on other risk, not solely medication side-effects and risk of driving; (f) ensuring that healthcare professionals have undergone appropriate training on how to discuss risks and (g) offering follow-up or interventions to those identified as high risk. The initial version of the guideline presented to participants included only recommendations 1–5; however, through the co-design process, participants highlighted the importance of incorporating structured risk communication training and the need for implementing additional follow-up consultations. These additions were subsequently integrated into the final guideline. Participants found the guideline informative, clear, enjoyed the design and further suggested embedding it into risk communication training.

A written leaflet (Appendix 3) was additionally developed to support patients and caregivers in retaining information communicated in face-to-face appointments. In addition to presenting risk information in a ‘doughnut’ display, which was most favoured by PPIE participants during the workshops, it also included simple wording for risk information such as ‘high risk’ and ‘top 10%’. Additionally, the leaflet contains information on the symptoms one may experience and what resources they can access when they need urgent support. Earlier versions of the leaflet provided limited detail on the symptoms that may require admission, offering only a brief mention of behaviours that may lead to distress. It was also lacking clear guidance for individuals identified as high risk. In the final co-design workshop, participants emphasised the importance of including more specific indicators of psychiatric crisis, along with practical strategies for response to those events and clearer pathways for accessing appropriate support, beyond general telephone contact details or website links.

## Discussion

This study explored how to best communicate the risk of future psychiatric admissions and needing enhanced crisis care to PLWD, caregivers and healthcare professionals, using RTA of co-design workshops. The materials discussed in the workshops, including the leaflet and the concept of using an app, were presented as preliminary or prototype solutions to stimulate discussion and begin further, necessary work on risk communication in the context of dementia.

Our findings suggest that participants place significant importance on the timing and setting of risk communication, supporting a shared and collaborative discussion of risks. Risk communication was perceived as complex and emotionally challenging, especially without certainty of risk reduction strategies. There was a strong preference for risk information to be delivered in a personalised manner, tailored to each individual, supported by a combination of visual, audio, written and digital tools to ensure it was accessible to those with sensory impairment. There was a preference for simple and clear communication, where familiarity and flexibility were valued.

In line with our work, other research has also shown that opinions and views of PLWD may often be overlooked by healthcare professionals.^25^ Although caregiver participation in consultations has been shown to enhance understanding and satisfaction, it can also shape the course of discussions, sometimes excluding or dominating the voices of the person with dementia, because of their perceived cognitive impairment.^7,26^ In terms of the workshops themselves, focus groups may make it easier for PLWD and their caregivers to participate together, but they can also limit the disclosure of personal experiences because of concerns about confidentiality among other participants or discomfort discussing sensitive issues (e.g. aggression, disinhibition) in the presence of the person they care for.^27^ Although many PLWD prefer to attend consultations and focus groups alongside their caregivers, this arrangement can sometimes limit the disclosure of certain information.

Previous research in this area has highlighted the lack of resources and information available on how to best communicate risk without any strategies available to support those at elevated risk.^5,6^ Similarly to our findings, a recent systematic review highlighted the need for additional consultation time allocated to those at high risk or delivering risk information at follow-up appointments.^28^ Continuity of care and trust in physicians were important aspects to participants throughout the workshop, and many feared being discharged from secondary services. Perhaps future strategies should work on implementing follow-up as part of primary care, with GPs and community services taking a more important role in the management of dementia. Continuity of care by GPs has been shown to lower rates of major adverse effects, result in safer prescribing and overall benefit for PLWD.^29^ However, given the current pressure on primary care, this may not be feasible, and alternatively it may be continuity in secondary care that could decrease the risk of care crises.

Another important aspect raised throughout the workshops and throughout feedback discussions on educational materials was the lack of risk mitigation strategies for people found at high risk. For many participants, despite acknowledging the importance of knowing what lies ahead of them, communicating risk without support seemed counterproductive, and many highlighted the crucial need for a targeted intervention to reduce risk. When asked about future directions, both the healthcare professional and patient and caregiver groups urged the focus to shift toward finding interventions to help prevent unplanned admissions. Future research should focus on finding solutions to manage the possibility of deterioration and introducing early access services targeted at reducing risk. However, it is also the case that knowing one is high risk might change behaviour, such as putting a power of attorney in place or creating a robust ‘what if’ plan should something happen to a carer.

### Strengths and limitations

Key strengths of this study include involving PLWD, carers and healthcare professionals in understanding communication needs and the co-design of educational tools. This enhances the acceptability, relevance and utility of the workshop’s outputs. The use of RTA allowed for active researcher input to the interpretation of the data and the integration of both semantic and latent meanings of the data. Multistakeholder perspectives allowed for a broader understanding of risk communication and contributed to the strengthening of our findings.

Our study has some limitations. A key limitation of this study relates to the small and relatively homogenous sample, which restricted the breadth of perspectives captured. Participants were approached on a voluntarily email basis, and we were unable to assess reasons for declining participation, but some may relate to limited availability, digital barriers and difficulty in participation because of cognitive difficulties. We aimed to recruit a diverse sample of participants in both socioeconomic and ethnic backgrounds; however, we were unable to do so, with underrepresentation of minoritised groups and PLWD with more advanced impairment. Although our study had a clear and focused aim (to inform early stage co-design of risk communication tools) and recruited participants with highly relevant lived and professional experience, the limited diversity of the sample constrained the richness of the insights. The themes generated should be regarded as exploratory and hypothesis-generating, providing direction for further participatory research involving larger and more diverse groups. Additionally, digital delivery of workshops may have excluded individuals less familiar with technology, such as those with more severe dementia. This also means that our sample of participants may have preferred a digital means of communicating risk over more traditional approaches, as they felt at ease using technology, which may not be applicable to all people with dementia.

In conclusion, this exploratory co-design study highlights the importance of personalised, well-timed and collaborative risk communication in dementia care. It provides early insights into communication about future risks of unplanned psychiatry admission, as well as more general principles of risk communication in this context of dementia. Co-designed tools, such as guidelines and patient leaflets, can support sensitive and transparent conversations, particularly when integrated with follow-up care and appropriate interventions. Further research with larger, more diverse samples is required to refine these approaches and to evaluate their impact in practice, alongside rigorous development and testing of any digital tools and, crucially, the development of interventions to support people at high risk of experiencing crises in their care.

## Supporting information

Swirska et al. supplementary material 1 Swirska et al. supplementary material

Swirska et al. supplementary material 2 Swirska et al. supplementary material

## Data Availability

The data that support the findings of this study are available on request from the corresponding author (M.S.). The data are not publicly available due to their containing information that could compromise the privacy of research participants.

## Supplementary material

The supplementary material is available online at https://doi.org/10.1192/bjo.2026.11011

## Appendix

**Appendix I:** Participant demographic table

Participant identifier	Gender	Living experience of dementia	Ethnicity	Educational background
Participant 1	Female	Carer (spouse)	White British	Higher education
Participant 2	Female	Carer (spouse)	White British	Higher education
Participant 3	Female	Carer (spouse)	White British	Higher education
Participant 4	Male	Person living with dementia	White British	Higher education
Participant 5	Male	Carer (spouse)	White British	Higher education
Participant 6	Male	Person living with dementia	White British	Higher education
Participant 7	Male	Psychologist	Asian or Asian British – Indian	Higher education
Participant 8	Female	Nurse	Black or Black British – African	Higher education
Participant 9	Female	Nurse	White British	Higher education
Participant 10	Female	Psychologist	White British	Higher education
Participant 11	Female	Doctor	Asian or Asian British – Pakistani	Higher education
Participant 12	Male	Doctor	Black or Black British – African	Higher education
