# Supplementary material for: Communicating the risk of psychiatric in-patient or enhanced community care in dementia
Source: BJPsych Open. 2026 Apr 7;12(3):e102. doi: 10.1192/bjo.2026.11011 (PMC13107325; doi:10.1192/bjo.2026.11011)
Supplement: Swirska et al. supplementary material 1 — Swirska et al. supplementary material [file S2056472426110114sup001.pdf]

Communicating risk in dementia care consultations: Recommendations for healthcare professionals

Aim: To provide clinicians with structured recommendations on risk communication to enhance patient understanding and support informed decision making.

Why should healthcare professionals communicate risk to people living with dementia?

- Supports patient autonomy
- Builds trust in healthcare professionals
- Allows for shared decision making between patients and clinicians
- Reduces anxiety and uncertainty
- Allows family members and caregivers to be involved
- Helps manage the risk in the future and make long-term plans

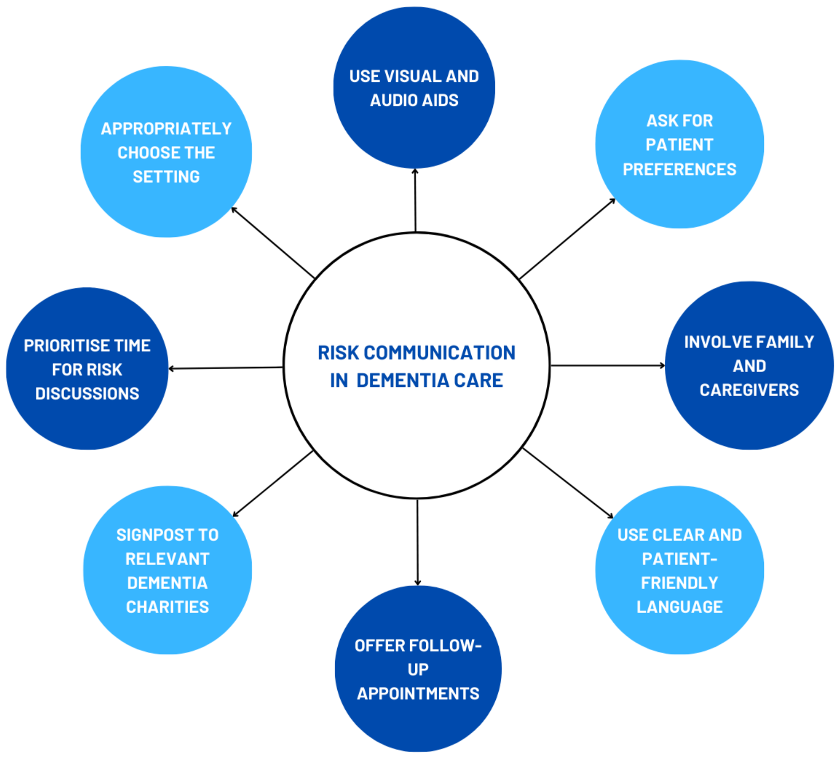

Recommendation 1- Risk communication should be clear, sensitive, and include regular checks for understanding.

Information regarding risk should be communicated clearly and concisely. Healthcare professionals should use straightforward language that reflects the way patients themselves speak, avoiding medical jargon. Healthcare professionals should break down information into appropriate chunks

and frequently check for understanding throughout the conversation. Discussing risk can be emotionally challenging, and healthcare professionals should ensure that they are having those conversations with empathy.

Recommendation 2- Risk communication should be tailored to each individuals' cognitive abilities, personal circumstances and emotions.

Risk information should be personalised to each patient, taking into account their cognitive abilities. A standardised approach may not be suitable for everyone, so healthcare professionals should always remain flexible in how they approach risk conversations. Because of the sensitive nature of risk communication, patients' personal circumstances, background, and emotions must be taken into consideration. Patients and carers value knowing their healthcare professionals before disclosing sensitive information, therefore it is important to build trust.

Recommendation 3- Family members and caregivers should be actively involved in risk-related conversations.

Family members and carers are recognised by both patients and healthcare professionals as vital to supporting individuals living with dementia. Their involvement in risk discussions can help in understanding, improve satisfactions with consultation, and additionally ensure that patient's values are respected, particularly in situations where patients may struggle to make decisions for themselves. Caregivers and family members may also help with communicating and implementing risk mitigation strategies.

Recommendation 4- A range of visual and audio aids should be offered to enhance patient understanding.

Visual and audio resources should be offered to support patients during risk communication. Visual aids should be adjusted to each individual's cognitive abilities. Patients and carers highlighted a preference for simple and effective formats such as pie charts or bar graphs, while more complex visuals, such as line graphs or risk array icons, may be useful for those with a better understanding of statistical information. For individuals with visual impairments, visual tools alone may not be sufficient, and, in this case, audio or video resources should be provided as alternative means of communication. These tools should also be offered as take-home materials.

Recommendation 5- Risk discussions in memory clinics should extend beyond driving and medication side effects.

Patients and caregivers rarely recall being informed about risks during their consultations. When risk was discussed, it was often limited to topics such as driving and medication side effects. While these issues remain important and should continue to be addressed, healthcare professionals should also explore a broader range of risks relevant to people with dementia. These include home safety, financial vulnerability, physical health concerns, and the risk of wandering or becoming lost. Additionally, discussions should consider the possibility of crisis events, a rapid deterioration in health, which may require urgent hospital or community interventions.

Recommendation 6- Healthcare professionals should receive training in risk communication and should prioritise time to discuss risk during consultations.

Healthcare professionals should receive structured training in risk communication, including role-playing exercises or simulated consultations, to help them in having those difficult consultations. An adequate amount of time should be dedicated to discussing risks and mitigation strategies in clinics. Brief and rushed conversations can make patients confused and may lead to misunderstandings. Effective communication builds trust and rapport, making patients and caregivers feel safe allowing them to raise any concerns.

Recommendation 7- Patients identified as being at higher risk should receive follow-up appointments to monitor and manage concerns.

Patients identified as being at higher risk should be offered follow-up appointments to monitor and manage ongoing concerns. Receiving information about an increased likelihood of dementia and its risks can be distressing for both patients and carers and providing them with additional support through regular follow-up can help reduce anxiety. These follow-up appointments will allow patients to feel supported, will ensure that future care plans and risk strategies are up-to-date, and will give clinicians to opportunity to offer more guidance and advice.
