# Supplementary material for: Communicating the risk of psychiatric in-patient or enhanced community care in dementia
Source: BJPsych Open. 2026 Apr 7;12(3):e102. doi: 10.1192/bjo.2026.11011 (PMC13107325; doi:10.1192/bjo.2026.11011)
Supplement: Swirska et al. supplementary material 2 — Swirska et al. supplementary material [file S2056472426110114sup002.pdf]

Reducing Your Risk of Needing Admission to Hospital

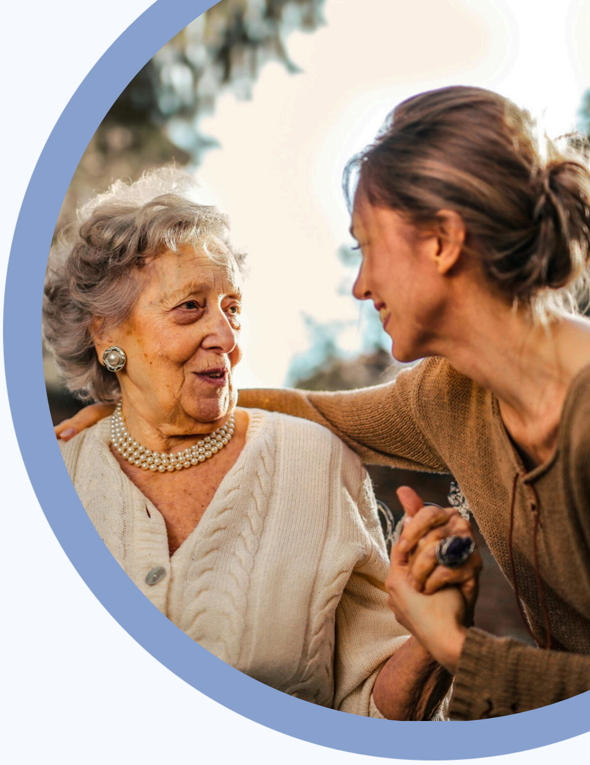

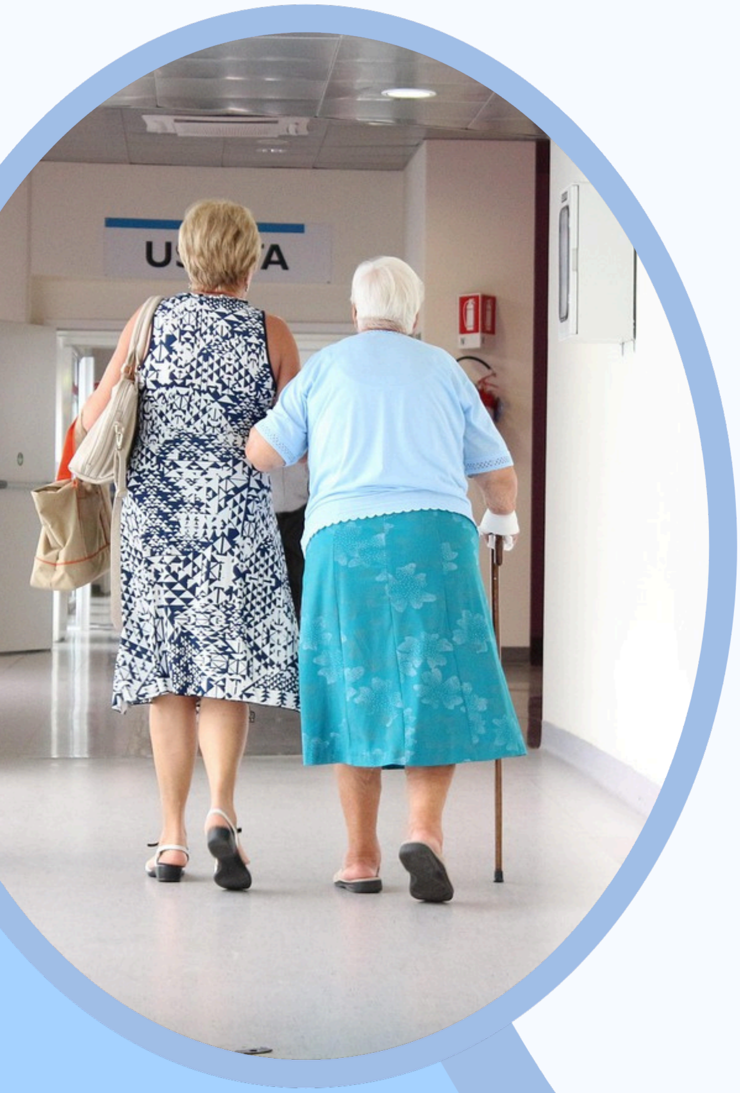

NEXT STEPS CHECKLIST

- Care Needs Assessment ✓
- Carer Assessment ✓
- Getting a PoA ✓
- Home Adjustments ✓
- Useful Contacts ✓

HELPING TO INTERVENE EARLY FOR BETTER OUTCOMES

HOW YOU CAN PREVENT THE NEED FOR EMERGENCY CARE

What is a Crisis event?

Sudden deterioration in health of people living with dementia at the point that they may require **urgent community support or hospital admission**

How Distress Can Become Apparent and Trigger the Need for Crisis Care:

- Becoming more confused or anxious
- Wandering off, getting lost
- Experiencing aggressive behaviours
- Experiencing hallucinations
- Not continuing with normal day to day events
- Patient or carer may struggle to cope

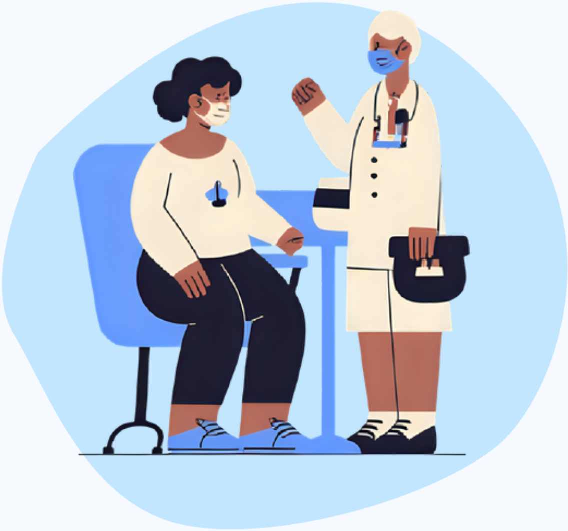

YOUR PERSONALISED RISK

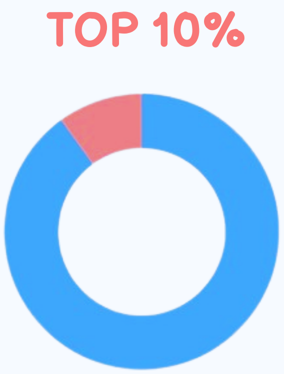

You are in the highest risk group (top 10%) of people living with dementia who are more likely to experience a change in your condition in the next year.

This means your may need extra support including enhanced community care or hospital admission.

What are your next steps?

1

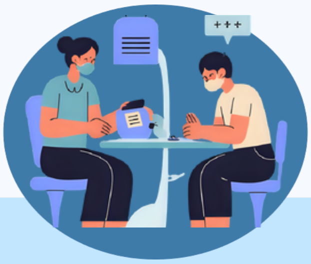

Care Needs Assessment

Patients can receive: assistance with social care such as:

- Bathing and dressing
- Meal delivery
- Financial assistance

2

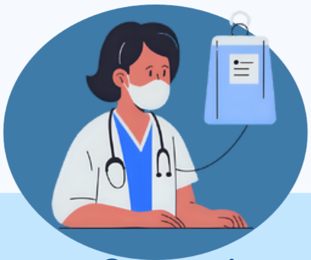

Carer's Assessment

Carers can receive:

- Caregiver breaks
- Transportation assistance
- Support groups and exercise classes
- Creation of What-if Plan
- Carer's Together Charity Support

3

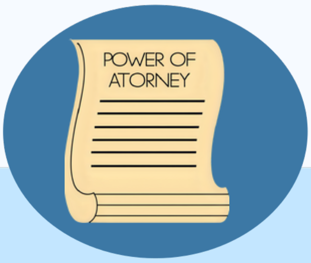

Getting a Power of Attorney

For patients who no longer have mental capacity, a PoA can legally make decisions about a patient's health needs, life prolonging treatment, and finances.

4

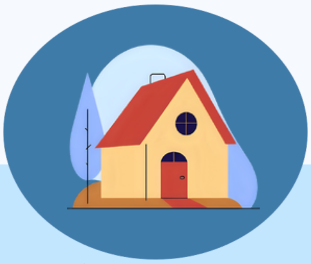

Home adjustments

A dementia friendly home includes:

- More natural light
- Noise reduction
- Flat surfaces and handrails
- Reminders or signs
- Community Occupational Therapy

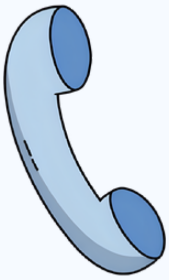

NHS mental health **111 option 2**
Dementia UK helpline: **0800 888 6678**
Dementia Support Line: **0333 150 3456**

Alzheimer's Society Website:
https://www.alzheimers.org.uk/
